# Prevalence of psychiatric vulnerability and neurocognitive disorders in nursing homes: impact on care levels

**DOI:** 10.1007/s41999-025-01179-y

**Published:** 2025-03-13

**Authors:** Katrin Gillis, Hanne Van Herbruggen, Marianne De Witte, Lore Baeck, Melanie Elisabeth Benoit Van Bogaert, Hilde Lahaye, Linda van Diermen

**Affiliations:** 1 Centre for Research and Innovation in Care, Antwerp University, Universiteitsplein 1, 2650 Wilrijk, Belgium; 2 Odisee University College, Centre for Health for People, Hospitaalstraat 23, 9100 Sint-Niklaas, Belgium; 3 Department of Psychiatry, Collaborative Antwerp Psychiatric Research Institute (CAPRI), Faculty of Medicine and Health Sciences, Antwerp University, Campus Drie Eiken, Universiteitsplein 1, 2610 Wilrijk, Belgium; 4 Odisee University College, Centre for Health for People, Hospitaalstraat 23, 9100 Sint-Niklaas, Belgium; 5 Vzw Curando, Pensionaatstraat 8A, 8755 Ruiselede, Belgium; 6 Psychiatric Center Bethanië, Andreas Vesaliuslaan 39, 2980 Zoersel, Belgium

**Keywords:** Dementia, Substance abuse, Depression, Anxiety, Nursing homes

## Abstract

**Purpose:**

There is an increasing number of residents with more complex needs in nursing homes. Due to the deinstitutionalisation of mental health care, more individuals with psychiatric vulnerabilities are being referred to nursing homes. The aim of this study is to gain insight into the prevalence of psychiatric vulnerability in Belgian nursing homes and its impact on care levels.

**Methods:**

After screening 3238 patient files of residents in twenty-four Belgian nursing homes, informed consent was obtained from 1155 of the 1608 residents or their legal representatives with a neurocognitive and/or psychiatric diagnosis. Residents were classified into three groups: residents with only a psychiatric diagnosis, with only a neurocognitive diagnosis, and both a psychiatric and neurocognitive diagnosis. The Health of Nations Outcome Scale 65 + was used to assess residents’ behaviour, limitations, symptoms, and functioning.

**Results:**

Of all residents, 17.5% had a lifetime psychiatric diagnosis and 41.8% had a neurocognitive disorder. Most prevalent were depressive disorder (8.2%) and Alzheimer’s disease (19.3%). Scores for behavioural problems (1.4 and 1.4 versus 0.9, *p* < 0.0001) and symptoms (5.5 and 5.1 versus 4.4, *p* < 0,0001) were higher in residents with only a psychiatric or both diagnoses compared to those with only neurocognitive disorders. Conversely, scores for limitations were higher in residents with only a neurocognitive disorder (3.6 versus 2.2 and 3.1, *p* < 0.0001).

**Conclusion:**

With almost 1 in 5 nursing home residents having a psychiatric vulnerability with higher levels of symptoms and behavioural problems, more attention towards improving nursing home caregivers’ competence in psychiatric care is recommended.

## Key summary points

**Aim:**

To gain insight on the prevalence of psychiatric vulnerability in Belgian nursing homes and its impact on care levels.

**Findings:**

Seventeen and a half percent of the residents have at least one documented lifetime psychiatric diagnosis and in 41.8% a neurocognitive disorder was documented. Residents with a psychiatric vulnerability scored higher on symptoms and behavioural problems compared to older adults with only a neurodegenerative disorder.

**Message:**

It is crucial to invest in nursing home staff training and education to enhance their competencies in the care of psychiatric vulnerable older adults.

## Background

The absolute number of residents in nursing homes is growing due to aging of the population. In 2010, about 60.000 older adults (65 +) lived in Flemish nursing homes, and this number increased up to 70.000 people by 2021. However, the overall percentage of older adults living in a nursing home decreased from 5.4% in 2010 to 5% in 2021 due to population growth. The mean age of nursing home residents was 87 in 2021, an increase of about one year since 2010. Eighty-four percent of those residents had high levels of care needs in 2021, representing a 14% increase compared to 2010. This implicates an enormous increase of pressure on nursing homes to provide the best care [1].

In recent years, the mental health care system in Belgium has been reorganised. There has been a shift towards deinstitutionalisation and a focus on mobile care teams, with a decrease in long-term psychiatric hospitalisation options and the possibility for people with serious psychiatric vulnerabilities to live in an adapted psychiatric care facility [2]. Consequently, more older adults are being directed to nursing homes following diagnosis, stabilisation, and treatment in a psychiatric hospital.

Healthcare providers also notice an increase in the number of residents with a psychiatric vulnerability in nursing homes [3]. Dealing with psychiatric vulnerability in this setting is challenging and when staff are not adequately educated to provide adapted care, problems could arise. Underdiagnosis and inadequate treatment of certain psychiatric disorders, such as major depressive disorder (MDD), could occur due to the normalisation of depressive symptoms [4].

A systematic review from Canada found a prevalence of major depressive disorder (MDD) of 10% among nursing home residents, while nearly 30% had depressive symptoms [5]. A meta-analysis in Italy estimated MDD prevalence in non-dementia residents at 18.9%, though attempts to estimate schizophrenia and bipolar disorder prevalence were hindered by insufficient data [6]. A study in Australia reported a 5% prevalence of anxiety disorders in nursing homes [7]. The handbook of Mental Health and Aging suggest that over half of those in assisted living facilities suffer from depressive disorders, with one-fifth newly diagnosed within their first year, raising concerns about depression’s impact on rehabilitation and mortality [8]. Dementia prevalence also varies across regions: in Canada, it was found to be 58% [5], while in Scotland, it was 58% with an additional 31.8% showing symptoms without a diagnosis [9]. In London, it reached 77%, alongside a 29.6% depression rate [10]. Mild cognitive impairment (MCI) affects around 21.2% of older adults in nursing homes globally [11]. Common neuropsychiatric symptoms in dementia include apathy (49%), depression (42%), aggression (40%), anxiety (39%), and sleep disorders (39%) [12]. A recent study of 13,413 dementia patients across Germany found agitation, aberrant motor behaviour, and irritability to be the most frequent behavioural and psychological symptoms of dementia (BPSD) [13]. Although individuals in this sample were diagnosed with diverse types of dementia, similarities in BPSD were found.

Care levels for older adults with dementia obviously increase when more BPSD are present [14]. It is reasonable to assume that the presence of neuropsychiatric symptoms also affects care levels. However, there is limited information in the literature about the specific levels of care needs of patients with a psychiatric vulnerability living in nursing homes. Managing the specific care needs of patients with either a neurocognitive disorder, a psychiatric disorder, or both presents additional challenges. Therefore, it is crucial to gain a better understanding of the prevalence of neurocognitive and psychiatric disorders—and by extension, psychiatric vulnerability—in nursing homes, and, moreover, the impact of these conditions on care intensity. The aim of this study is to provide a clear overview of the levels of care for older adults with psychiatric vulnerabilities. By mapping the prevalence rates, the overall impact on care in nursing homes can be outlined. *What is the prevalence of psychiatric vulnerability and neurocognitive disorders amongst nursing home residents in Belgium and what is its impact on care levels?*

## Materials & methods

### Study design and population

A quantitative cross-sectional study was conducted between January 2022 and September 2022 in Belgian nursing homes. A stratified sample of eighty nursing homes was selected, with stratification based on geographical location, number of residents and type of organisation (public or non-public). The research protocol was submitted to and approved by the ethical committee of the University Hospital of Antwerp (B3002021000188). The electronic patient records of nursing homes willing to participate were evaluated to determine the prevalence of neurocognitive or psychiatric disorder according to the Diagnostic and Statistical Manual of Mental Disorders (DSM-V) criteria. Residents or their legal representatives (in case of cognitive impairment) with a diagnosed psychiatric or neurodegenerative disorder were invited to participate in the study. Data could only be collected after obtaining informed consent. The nursing home staff approached residents or their legal representatives to obtain their informed consent.

### Assessments

Demographic data, such as age, gender, duration of residence, civil status, medication scheme, score on the Mini-Mental State Examination (MMSE), medical history, and the Katz index were extracted from patient records. Age and duration of residence were measured in years. Gender was recorded as male or female. Civil status included categories single, married, widowed, or divorced. The medication scheme covered the use of psychotropic drugs, such as benzodiazepines, antidepressants, antipsychotics, mood stabilizers, anti-Parkinson drugs, anti-Alzheimer drugs and opioids.

The MMSE is a widely used tool for assessing cognitive function. It consists of 11 questions that evaluate key areas like orientation, memory recall, attention, and language, with a total score ranging from 0 to 30—where higher scores indicate better cognitive functioning. For the purpose of analysis, scores were divided into four categories: category 1 includes scores below 10, category 2 ranges from 10 to 18, category 3 covers scores between 18 and 24, and category 4 includes scores above 24. Psychometrically, the MMSE has demonstrated moderate to high internal consistency, with Cronbach’s alpha values between 0.60 and 0.90, depending on the population and context. It has also shown strong validity in detecting cognitive impairments, such as dementia, making it a standard tool in geriatric assessments [15].

The Katz index of Independence in Activities of Daily Living (ADL) categorizes residents into six levels based on their ability to perform basic tasks such as bathing, dressing, and eating. The categories range from O, indicating full independence, to D, indicating severe dependence. The intermediate categories (A, B, C, Cd) represent varying levels of dependence, with Cd indicating both physical and cognitive impairments [16]. The Katz Index has demonstrated good internal consistency, Cronbach’s alpha values ranging from 0.84 to 0.94, and good inter-rater reliability, with kappa values between 0.87 and 0.92. This measure is well-validated for assessing the functional independence of older adults in activities of daily living [17].

The Dutch version of the Health of Nations Outcome Scale 65 + (HoNOS 65 +) is a validated instrument designed to evaluate the psychiatric, functional, social, and somatic functioning of older adults using a Likert scale (0 = no problem, 4 = severe problem) [18, 19]. It comprises four subscales with twelve elements, including behavioural problems, intentional self-harm, alcohol or drug abuse, cognitive problems, problems due to somatic limitations of illnesses, hallucinations or delusions, depressive symptoms, other mental or behavioural problems, social problems due to personal relationships, problems with activities of daily living, and the quality of daily routine. Residential problems were not evaluated, since all participants already lived in a nursing home. In total, 45 items were scored.

Psychometrically the reliability of HoNOS 65 + has shown acceptable inter-rater reliability, with studies reporting kappa values ranging from 0.69 to 0.89. Its validity is demonstrated through its ability to differentiate between various severity levels of psychiatric conditions, and its internal consistency is typically moderate, with Cronbach’s alpha values around 0.70. The scale is widely used to assess psychiatric, social, and functional domains in older populations [20].

When classifying behavioural problems, a continuous variable, HoNOS 65 + scores below 0.5 were categorised as having no significant behavioural problems. Scores including 0.5 but below 2 were categorised as mild to moderate symptoms, while scores of 2 or higher were considered indicative of severe behavioural problems. For depressive symptoms, a score of 0 indicated no symptoms, scores of 1 or 2 were classified as mild to moderate symptoms, and scores of 3 or 4 as severe depressive symptoms.

### Data collection

After the nursing home staff obtained informed consent, three researchers visited the different nursing homes. Caregivers were responsible for collecting the demographic data. All researchers completed an online course to become a certified HoNOS 65 + assessor. They filled in the HoNOS 65 + by interviewing a caregiver who was familiar with the situations of the residents with informed consent. Based on the caregiver’s responses, the researchers completed the questionnaire.

### Statistical methods

Prevalence rates of neurocognitive and psychiatric disorders were calculated and extrapolated to the total of residents with a correction for residents with a confirmed diagnosis who refused participation (1608/1155 = 1.39 (n × 1.39)). The residents were classified into three groups, based on medical written diagnoses in their records: older adults with psychiatric vulnerability, older adults with a neurocognitive disorder, and older adults with both psychiatric vulnerability and a neurocognitive disorder. To compare care levels as measured by the HoNOS 65 + , a one-way analysis of covariance (ANCOVA) was conducted using IBM SPSS Statistics Version 29.0.2.0. Where significant differences between groups were found, Tukey’s honestly significant difference test was used to identify which groups differed from one another.

## Results

### Prevalence

**Fig. 1:**
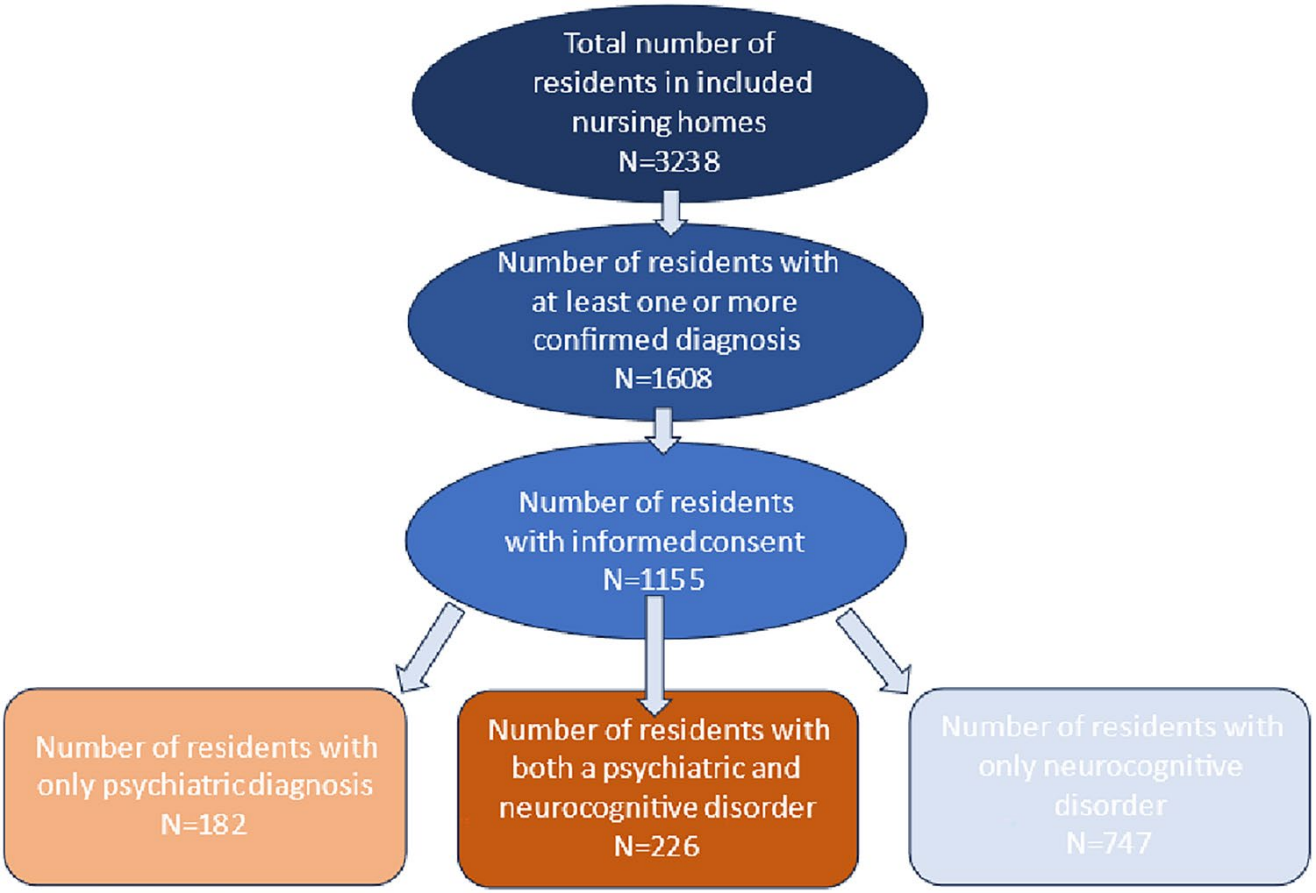
Participant flow

A total of twenty-four public and non-public nursing homes, with a minimum of 91 and a maximum of 211 older adults, participated in the study. All nursing homes were located in Flanders, the Northern part of Belgium (See additional file). Altogether, 3238 residents lived in these nursing homes, of whom 1608 (49.7%) had one or more confirmed diagnoses of a psychiatric or a neurocognitive disorder. Out of those with a confirmed diagnosis, 1155 agreed to participate (Fig. 1). After extrapolation, seventeen and a half percent (*n* = 568) of all residents had at least one documented lifetime psychiatric diagnosis, indicating increased psychiatric vulnerability. In 41.8% (*n* = 1354) of residents, a neurocognitive disorder was documented.

### Demographic characteristics

The research population as shown in Table 1 contained more women (72.4%) than men (27.6%). Residents with a psychiatric vulnerability (80.7 years) or both a psychiatric and a neurocognitive disorder (81.3 years) were significantly younger than those with only a neurocognitive disorder (86.4 years). Those with a psychiatric vulnerability had the longest duration of residence (4.8 years), while those with only a neurocognitive disorder had the shortest (2.7 years). Residents with only a psychiatric vulnerability predominantly lived in open wards (78.6%). Overall, residents with only a psychiatric vulnerability have a lower Katz category compared to those with a neurocognitive disorder or both disorders.

**Table 1:** Description of the research population

	Research groups				
	Total (*n* = 1155)	Only neurocognitive disorder (*n* = 747)	Only psychiatric disorder (*n* = 182)	Both (*n* = 226)	*p*-value
Gender					
Male	318 (27.6)	182 (24.4)^a^	58 (31.9)^ab^	78 (34.5)^b^	
Female	836 (72.4)	564 (75.6)^b^	124 (68.1)^ab^	148 (65.5)^a^	
Age (yrs), mean (± SD)	84.5 (± 8.1)	86.4 (± 6.7)^b^	80.7 (± 8.6)^a^	81.3 (± 9.8)^a^	< 0.001
Duration of residence (yrs), mean (± SD)	3.3 (± 3.9)	2.7 (± 2.9)^a^	4.8 (± 6.2)^c^	3.7 (± 3.25)^b^	< 0.015
Ward type					
Secluded	444 (38.4)	318 (42.6)^b^	39 (21.4)^a^	87 (38.5)^b^	< 0.001
Open	711 (61.6)	429 (57.4)^a^	143 (78.6)^b^	139 (61.5)^a^	< 0.001
Use of psychotropic drugs					
Benzodiazepines and other sleep medication*	489 (42.3)	283 (37.9)^a^	96 (52.7)^b^	110 (78.7)^b^	< 0.001
Antipsychotics	474 (41.0)	283 (37.9)^a^	87 (47.8)^b^	104 (46.0)^b^	0.001
Antidepressants	418 (36.2)	218 (29.2)^a^	91 (50.0)^b^	109 (48.2)^b^	< 0.001
Antiepileptics and mood stabilizers	135 (11.7)	55 (7.4)^a^	37 (20.3)^b^	43 (19.0)^b^	< 0.001
Anti-Parkinsonian	140 (12.1)	82 (11.0)	25 (13.7)	33 (14.6)	0.541
Anti-Alzheimer	147 (12.7)	122 (16.3)	5 (2.7)^a^	20 (8.8)^a^	< 0.001
Opioids	163 (14.1)	32 (17.6)	93 (12.4)	38 (16.8)	0.176
Katz total (8–32), mean (± SD)	23.0 (± 5.5)	23.9 (± 5.2)^c^	19.7 (± 5.2)^a^	22.6 (± 5.6)^b^	< 0.001
Category-O	30 (2.6)	7 (0.9)^a^	18 (9.9)^b^	5 (2.2)^a^	< 0.001
Category-A	106 (9.2)	41 (5.5)^a^	49 (26.9)^b^	16 (7.2)^a^	< 0.001
Category-B	340 (29.5)	220 (29.5)	51 (28.0)	69 (30.9)	0.831
Category-C	100 (8.7)	52 (7.0)^a^	29 (15.9)^b^	19 (8.5)^a^	< 0.001
Category-Cd	508 (44.1)	378 (50.7)^b^	33 (18.1)^a^	97 (43.5)^b^	< 0.001
Category-D	65 (5.6)	47 (6.3)^b^	1 (0.5)^a^	17 (7.6)^b^	0.004
MMSE (0–30), mean (*n* = 767) **(**± SD)	16.5 **(**± 7.3)	14.9 **(**± 7.0)	20.9 **(**± 7.0)	18.1 **(**± 6.8)	< 0.001

Values are presented as counts, *n* (%) unless indicated otherwise

^abc^Values that do not share the same superscript on the same line are statistically significantly different after Tukey adjustment for multiple comparisons

*Z-drugs and trazodone

**Fig. 2:**
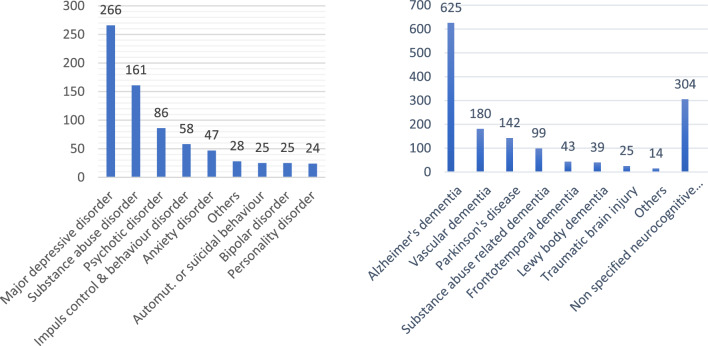
**A** Distribution of types of psychiatric disorder (total *n* = 408) and **B** neurocognitive disorders (total *n* = 973) Some residents have multiple diagnoses and are therefore counted multiple times in this figure

The most prevalent psychiatric disorder was MDD, recorded in 5.9% of all residents of the participating nursing homes. (8.2% when extrapolated to include those who did not give consent). Substance abuse disorder was the second most common, followed by psychotic disorders, affecting 3.6% (5.0%) and 1.9% (2.7%) of all residents, respectively (Fig. 2). Of all residents, 0.7% (1.4%) was diagnosed with an anxiety disorder.

Among neurocognitive disorders, Alzheimer’s disease is the most prevalent, with 13.9% (19.3%) of all residents having a documented neurocognitive diagnosis, followed by vascular dementia and Parkinson’s disease dementia, affecting 3.9% (5.6%) and 3.1% (4.3%) of residents, respectively.

### Behavioural problems

The presence of behavioural problems was also assessed. Residents with psychiatric vulnerability were compared to those with only a neurocognitive disorder and to those with both diagnoses (Table 2). Based on HoNOS 65 + scores, residents with only a psychiatric diagnosis (1.4) (or both, 1.4) had a significantly higher score for behavioural problems than residents with only a neurocognitive disorder (0.9, *p* < 0.0001). Of the residents with psychiatric vulnerability, 72.3% presented with some level of behavioural problems (*n* = 229). The majority (44.8%) had mild to moderate symptoms, while only 27.4% had severe symptoms (*p* < 0.001). There were fewer residents with a neurocognitive disorder who showed some level of behavioural problems, specifically 64.8% (*n* = 631). Among these, 50.4% had mild to moderate symptoms, and 14.5% had severe symptoms (*p* < 0.001).

**Table 2:** HoNOS 65 + mean scores in the three research groups (neurocognitive diagnosis, psychiatric disorder, both)

HoNoS 65 + item	Research groups			
	Only neurocognitive disorder (*n* = 747)	Only psychiatric disorder (*n* = 182)	Both (*n* = 226)	*p*-value
Behavioural problems (0–12)	0.9 (± 0.8)^a^	1.4 (± 1.5)^b^	1.4 (± 1.3)^b^	< 0.0001*
Limitations (0–8)	3.6 (± 1.4)^c^	2.2 (± 1.3)^a^	3.1 (± 1.5)^b^	< 0.0001*
Symptoms (0–12)	4.4 (± 2.3)^a^	5.5 (± 2.6)^b^	5.1 (± 2.4)^b^	< 0.0001*
Social problems (0–12)	6.1 (± 2.5)	5.8 (± 2.6)	6.0 (± 2.4)	0.4643
HoNOS Total score (0–44)	14.9 (± 5.7)	15.0 (± 5.2)	15.7(± 5.3)	0.2263

Values are presented as mean ± SD

^abc^Values that do not share the same superscript on the same line are statistically significantly different after Tukey adjustment for multiple comparisons

Residents with a psychiatric vulnerability alone, or both a psychiatric and a neurocognitive disorder, presented with higher symptom levels than those with only a neurocognitive disorder (5.5 and 5.1 versus 4.4, *p* < 0.0001). Conversely, residents with only a neurocognitive disorder had higher levels of limitations (3.6 versus 2.2 and 3.1, *p* < 0.001). There is a significant correlation between the Katz index and limitations as measured by HoNOS 65 + (*p* < 0.001).*.*

The most frequent behavioural problems are comparable across the three research groups, with restlessness being the most prevalent (58.2–65.5%), followed by agitation (53.5–61.5%), uncooperative attitude (50.5–54.9%), and aggression (42.3–50.4%). Important symptoms that impact daily functioning include depressive symptoms (54.5–78%) and cognitive distortions (44.5–47.8%). A total of 696 residents were assessed for depressive symptoms, of whom 277 had limited symptoms. Four hundred and nineteen residents had mild to severe symptoms (36.3%). When extrapolated to all residents, 18% are estimated to have clinically relevant depressive symptoms.

Table 3 compares the mean scores of the same behavioural problems and symptoms, revealing no significant differences between the research groups for most problems. The only significant differences are seen in restlessness, agitation, wandering, and bizarre behaviour, with the highest scores for residents with both diagnoses. Depressive symptoms were most severe in residents with a psychiatric disorder (1.9), followed by those with both disorders (1.52), and lowest in those with only a neurocognitive disorder (1.01; *p* < 0.001).

**Table 3:** Means of behavioral problems and symptoms measured with HoNOS 65 + scores in the three research groups (neurocognitive diagnosis, psychiatric disorder, both): results of the ANOVA analyses of outcome variables

Behavioural problems	Research groups			
	Only neurocognitive disorder (*n* = 747)	Only psychiatric disorder (*n* = 182)	Both (*n* = 226)	*p*-value
Overactive	0.7 (± 1.22)	0.7 (± 1.24)	0.89 (± 1.43)	0.136
Aggressive	0.86 (± 1.13)	0.8 (± 1.10)	1.02 (± 1.26)	0.101
Disruptive or destructive behaviour to persons or objects	0.47 (± 0.98)	0.55 (± 1.03)	0.61 (± 1.16)	0.164
Restlessness	1.34 (± 1.35)^a^	1.3 (± 1.40)^a^	1.68 (± 1.50)^b^	0.003*
Agitation	1.17 (± 1.27)^a^	1.34 (± 1.34)^b^	1.46 (± 1.38)^b^	0.007*
Uncooperative of resistive	1.22 (± 1.32)	1.1 (± 1.28)	1.23 (± 1.32)	0.552
Wandering	0.29 (± 0.79)^b^	0.14 (± 0.57)^a^	0.35 (± 0.84)^b^	0.02*
Inappropriate and disinhibited behaviour	0.51 (± 1.21)	0.65 (± 1.27)	0.71 (± 1.35)	0.074
Inappropriate vocalisation	0.52 (± 1.14)	0.55 (± 1.13)	0.67 (± 1.28)	0.212
Bizarre behaviour	0.18 (± 0.78)^a^	0.3 (± 0.98)^ab^	0.38 (± 1.04)^b^	0.008*
Delusions	0.7 (± 1.24)	0.86 (± 1.41)	0.73 (± 1.30)	0.352
Hallucinations	0.48 (± 1.07)	0.64 (± 1.24)	0.5 (± 1.12)	0.199
Cognitive distortions or thought disorder	1.13 (± 1.40)	1.13 (± 1.45)	1.25 (± 1.48)	0.544
Mood disturbance or depressive symptoms	1.01 (± 1.19)^a^	1.9 (± 1.39)^c^	1.52 (± 1.41)^b^	< 0.001*

Values are presented as mean ± SD

^abc^Values that do not share the same superscript on the same line are statistically significantly different after Tukey adjustment for multiple comparisons

In our total population, 8.2% (*p* < 0.001) have a lifetime diagnosis of MDD. Among residents with a diagnosed neurocognitive disorder, the prevalence of a MDD diagnosis is 10.8% (*p* < 0.001), while for those with only a psychiatric disorder, it is 47.2% (*p* < 0.001). However, 60.3% (*p* < 0.001) of residents in the research group had depressive symptoms based on HoNOS 65 + , with 40.2% having mild to moderate symptoms and 20.1% having severe symptoms.

## Discussion

This study aimed to assess the prevalence of neurocognitive and psychiatric disorders among residents in Belgian nursing homes and examine their care needs. The prevalence of neurocognitive disorders was 41.8%, while psychiatric disorders affected 17.5% of residents. In comparison, studies from the UK and Canada reported dementia rates in nursing homes ranging from 58 to 77%, with an additional 31.8% of residents exhibiting symptoms without a formal diagnosis [5, 9, 10]. The lower prevalence in Belgium may be due to the use of the Katz index for nursing home admissions, where a formal diagnosis of dementia is not always required unless the resident has a high level of physical independence.

Data in the literature on the prevalence of psychiatric disorders, aside from major depressive disorder (MDD), was limited. Substance use disorder was present in 5.0% of residents, psychotic disorders in 2.7%, and anxiety disorders in 1.8%. These numbers align with estimates from Canada, where anxiety disorders ranged from 3.5 to 11.7%, schizophrenia from 3.6%, and substance use disorder from 1.0 to 2.8%, with up to 18% of residents exhibiting symptoms of substance abuse [5].

The extrapolated prevalence of MDD in this study was 8.2%. This is lower than the 11.1–13.3% found in general populations of older adults in studies from Iran, Spain, and Brazil [21–23]. Another study in Brazil found that 25.6% of older adults showed clinically significant depressive symptoms. A systematic review and meta-analysis in Italy reported a prevalence of MDD among non-dementia nursing home residents at 18.9% [6]. In this study, using the HoNOS 65 + to measure depressive symptoms, a prevalence of 18% was found, aligning with Fornaro et al.’s findings. The underreporting of MDD in Belgian nursing homes may result from incomplete patient files and underdiagnosis, a problem highlighted in Spain by Gutiérrez-Rojas et al. [24]. Barriers to diagnosis include the masking of depression by chronic illness and pain and the normalization of depressive symptoms as part of aging [25, 26]. Additionally, older adults may be less likely to seek help, as shown in a study from Brazil [4].

There is significant symptom overlap between MDD and neurocognitive disorders, leading to frequent co-diagnoses. Proper diagnosis is crucial, as treatment significantly improves quality of life [27]. Non-pharmacological strategies, such as music and aromatherapy, are effective in treating MDD in older adults with dementia, according to a UK meta-analysis [28].

There is a knowledge gap concerning care levels in nursing homes. Research in Italy indicates that psychiatric vulnerability negatively impacts quality of life and functioning [29]. High scores on the HoNOS 65 + scale correspond with higher individual care levels [19]. Neurocognitive and psychiatric vulnerabilities often overlap, with residents who have both disorders showing the highest scores for behavioural issues. Agitation, improper behaviour, and other neuropsychiatric symptoms significantly increase care needs. Overall those behavioural problems and symptoms are more common in residents with psychiatric vulnerabilities and those with both a psychiatric vulnerability and a neurocognitive disorder. However, the Belgian care system, which relies on the Katz index for funding, does not account for these behavioural problems. Although overall scores for behavioural problems on a 12-point scale were low (0.9–1.4), their prevalence was high. Mild behavioural problems are common, which may downplay the scores. These scores do not reflect the clinical impact of the problem or its magnitude. In a clinical setting, the HoNOS 65 + can be used at the individual level by focusing on each item separately [19].

In this study, behavioural problems were present in 72.3% of residents with psychiatric disorders and 64.8% of those with neurocognitive disorders, with severe symptoms in 27.4% and 14.5%, respectively. These numbers align with other studies, such as those from Italy and France, which report behavioural and psychological symptoms of dementia (BPSD) rates of up to 96.4% in frontotemporal dementia and 90% in Alzheimer’s disease [30, 31]. These higher percentages reflect the behavioural problems that occur during the entire dementia process, from diagnosis to death, rather than a one-off sample from a differentiated group with varying stages of the disease. The severity and frequency of symptoms typically increase as dementia progresses. Different neurocognitive disorders are linked to distinct BPSD patterns [30, 32]. In this study, common behavioural symptoms included restlessness (60.9%), depressive symptoms (54.5%), agitation (53.5%), non-cooperation (54.9%), cognitive distortions (46.1%), and aggression (45.6%). While most studies focus on BPSD, little data exists on similar symptoms in residents with psychiatric disorders. A meta-analysis from Germany found agitation/aggression (36%), depression/dysphoria (33%), and apathy/indifference (33%) to be the most prevalent neuropsychiatric symptoms in long-term care residents with dementia [32]. However, differences in screening tools, such as the Neuropsychiatric Inventory – Nursing Home Version, make direct comparisons challenging.

This study highlights the need for more comprehensive psychiatric care in nursing homes, emphasizing the importance of addressing both neurocognitive and psychiatric disorders.

## Strengths and limitations

One of the strengths of this study is its large sample size, which allowed for a comprehensive analysis of participants’ daily functioning, well-being, and medication use. The detailed data enhance the reliability of the findings. However, future research could benefit from incorporating current psychiatric diagnoses instead of relying solely on electronic patient records, although this would be a significant challenge given the sample size.

A key limitation is the absence of baseline care data for residents without psychiatric or neurodegenerative diagnoses. Including such a control group through HoNOS 65 + assessments would provide insights into the care needs of residents with only physical or social issues. Another limitation is the potential for selection bias, as only residents who provided consent were included, possibly leading to an underrepresentation of more severe cases. The study was also limited to residents with confirmed diagnoses, raising the possibility of underdiagnosis affecting the sample. Finally, although all researchers followed the same guidelines and training, slight variations in interpreting signs and symptoms could have occurred.

Expanding our understanding of effective approaches for this vulnerable population is essential for improving care practices. Non-pharmacological interventions, such as music therapy, aromatherapy, and touch therapy, have shown promise in managing behavioural problems [33]. Need-based care has been particularly effective in addressing behavioural issues in dementia patients [34, 35]. This raises the question of whether it is necessary to distinguish between neurocognitive and psychiatric vulnerabilities, as both may require similar care levels but possibly different interventions.

Furthermore, this highlights the need for enhanced training for nursing staff, ensuring their skills align with the growing psychiatric needs of nursing home residents. Geriatric psychiatric outreach teams are increasingly being deployed to assist staff with particularly challenging cases. Strengthening collaboration between mental health professionals and nursing homes could further improve the quality of life for residents with psychiatric and/or neurocognitive disorders.

## Conclusions

With 17.5% of older adults in nursing homes having a lifetime psychiatric diagnosis and showing a higher score on symptoms and behavioural problems compared to older adults with only a neurocognitive disorder, these findings have significant clinical relevance. In addition to the existing training in dementia care, it is crucial to invest in staff training and education to enhance their competencies in psychiatric care. For neuropsychiatric symptoms in residents with dementia, need-based care has proven to be successful [34, 35]. However, limited research has been done on methods to improve psychiatric care competencies in nursing homes.

## Data Availability

The datasets used and analysed during the current study are available from the corresponding author upon reasonable request.

## References

[1] Statistics Flanders. (2023). Zorg en ondersteuning voor ouderen - Statistiek Vlaanderen. https://www.vlaanderen.be/statistiek-vlaanderen/zorg/zorg-en-ondersteuning-voor-ouderen

[2] Adriaenssens J et al (2018) Hoe de organisatie van de geestelijke gezondheidszorg voor ouderen verbeteren? 10.57598/R301AS

[3] Fedral Public Service (2021) Key data in healthcare: mental healtcare - edition 2021. https://www.healthybelgium.be/images/Blikvanger_Gezondheidszorg_GGZ_EN_v08.pdf

[4] Faisal-Cury et al (2022) Depression underdiagnosis: prevalence and associated factors. A population-based study. J Psychiatr Res 151:157–165. 10.1016/j.jpsychires.2022.04.025 35486997 10.1016/j.jpsychires.2022.04.025

[5] Seitz D, Purandare N, Conn D (2010) Prevalence of psychiatric disorders among older adults in long-term care homes: a systematic review. Int Psychogeriatr 22(7):1025–1039. 10.1017/S1041610210000608 20522279 10.1017/S1041610210000608

[6] Fornaro M et al (2020) Prevalence and correlates of major depressive disorder, bipolar disorder and schizophrenia among nursing home residents without dementia: systematic review and meta-analysis. Br J Psychiatry 216(1):6–15. 10.1192/bjp.2019.5 30864533 10.1192/bjp.2019.5

[7] Creighton AS, Davison TE, Kissane DW (2016) The prevalence of anxiety among older adults in nursing homes and other residential aged care facilities: a systematic review. Int J Geriatr Psychiatry 31(6):555–566. 10.1002/gps.4378 26552603 10.1002/gps.4378

[8] Hantke N, Etkin A, O’Hara R (2020) Handbook of Mental Health and Aging (3rd ed.).

[9] Lithgow S, Jackson GA, Browne D (2012) Estimating the prevalence of dementia: cognitive screening in glasgow nursing homes. Int J Geriatr Psychiatry 27(8):785–791. 10.1002/gps.278410 22081511 10.1002/gps.2784

[10] Stewart et al (2014) Current prevalence of dementia, depression and behavioural problems in the older adult care home sector: The South East London care home survey. Age Ageing 43(4):562–567. 10.1093/ageing/afu062 24855111 10.1093/ageing/afu062

[11] Chen et al (2023) Global prevalence of mild cognitive impairment among older adults living in nursing homes: a meta-analysis and systematic review of epidemiological surveys. Translat Psychiatry. 10.1038/s41398-023-02361-1 10.1038/s41398-023-02361-1 PMC10008549 36906613

[12] Zhao QF et al (2016) The prevalence of neuropsychiatric symptoms in Alzheimer’s disease: systematic review and meta-analysis. J Affect Disord 190:264–271. 10.1016/j.jad.2015.09.069 26540080 10.1016/j.jad.2015.09.069

[13] Schwertner et al (2022) Behavioral and psychological symptoms of dementia in different dementia disorders: a large-scale study of 10,000 individuals. J Alzheimer’s Disease 87(3):1307–1318. 10.3233/JAD-215198 35491774 10.3233/JAD-215198 PMC9198804

[14] Henskens M et al (2019) Predictors of care dependency in nursing home residents with moderate to severe dementia: a cross-sectional study. Int J Nurs Stud 92:47–54. 10.1016/j.ijnurstu.2018.12.005 30703703 10.1016/j.ijnurstu.2018.12.005

[15] Folstein MF, Folstein SE, McHugh PR (1975) “Mini-mental state”. A practical method for grading the cognitive state of patients for the clinician. J Psychiatr Res 12(3):189–98. 10.1016/0022-3956(75)90026-6 1202204 10.1016/0022-3956(75)90026-6

[16] RIZIV (2024) *Evaluationscale (Katz)*. https://www.riziv.fgov.be/nl/professionals/individuele-zorgverleners/verpleegkundigen/verstrekkingen-door-verpleegkundigen/evaluatieschaal-katz

[17] Health Belgium. (n.d.) Katz index of Independance in Activities of daily living. Retrieved October 28, 2024, from https://www.health.belgium.be/sites/default/files/uploads/fields/fpshealth_theme_file/katztext.pdf

[18] Harris MG (2023) Assessing the content validity of the revised Health of the nation outcome scales 65+: the HoNOS older adults. BJPsych Bull 47(4):195–202. 10.1192/bjb.2022.37 35916442 10.1192/bjb.2022.37 PMC10387420

[19] Veerbeek MA, Oude Voshaar RC, Pot AM (2013) Psychometric properties of the dutch version of the health of the nation outcome scales for older adults (HoNOS 65+) in daily care. Int J Nurs Stud 50(12):1711–1719. 10.1016/j.ijnurstu.2013.05.004 23768517 10.1016/j.ijnurstu.2013.05.004

[20] Burns et al (1999) Health of the nation outcome scales for elderly people (HoNOS 65+). Br J Psychiatry 174(5):424–427. 10.1192/bjp.174.5.424 10616609 10.1192/bjp.174.5.424

[21] Abdoli N et al (2022) The global prevalence of major depressive disorder (MDD) among the elderly: a systematic review and meta-analysis. Neurosci Biobehav Rev 132:1067–1073. 10.1016/j.neubiorev.2021.10.041 34742925 10.1016/j.neubiorev.2021.10.041

[22] Arias-de la Torre J et al (2021) Prevalence and variability of current depressive disorder in 27 European countries: a population-based study. Lancet Public Health 6(10):e729–e738. 10.1016/S2468-2667(21)00047-5 33961802 10.1016/S2468-2667(21)00047-5 PMC8460452

[23] da Costa L, Dias F et al (2019) Prevalence of late-life depression and its correlates in a community-dwelling low-educated population aged 75+ years: The Pietà study. J Affect Disord 242:173–179. 10.1016/j.jad.2018.08.012 30189354 10.1016/j.jad.2018.08.012

[24] Gutiérrez-Rojas L et al (2020) Prevalence and correlates of major depressive disorder: a systematic review. Revista Brasileira de Psiquiatria 42(6):657–672. 10.1590/1516-4446-2020-0650 32756809 10.1590/1516-4446-2019-0650 PMC7678895

[25] Baldwin RC (2000) Poor prognosis of depression in elderly people: causes and actions. Ann Med 32(4):252–256. 10.3109/07853890009011769 10852141 10.3109/07853890009011769

[26] Heok KE, Ho R (2008) The many faces of geriatric depression. Curr Opin Psychiatry 21(6):540–545. 10.1097/YCO.0b013e328311cdae 18852559 10.1097/YCO.0b013e328311cdae

[27] Sivertsen H et al (2015) Depression and quality of life in older persons: a review. Dement Geriatr Cogn Disord 40(5–6):311–339. 10.1159/000437299 26360014 10.1159/000437299

[28] Watt JA (2021) Comparative efficacy of interventions for reducing symptoms of depression in people with dementia: systematic review and network meta-analysis. BMJ (Clin Res Ed) 372:n532. 10.1136/bmj.n532 10.1136/bmj.n532 PMC7988455 33762262

[29] Grassi L et al (2020) Quality of life, level of functioning, and its relationship with mental and physical disorders in the elderly: results from the MentDis_ICF65+ study. Health Qual Life Outcomes 18(1):61. 10.1186/s12955-020-01310-6 32143635 10.1186/s12955-020-01310-6 PMC7060594

[30] Cognat et al (2023) BPSD patterns in patients with severe neuropsychiatric disturbances: insight from the RECAGE Study. Am J Geriatr Psychiatry 31(8):633–639. 10.1016/j.jagp.2023.03.014 37183097 10.1016/j.jagp.2023.03.014

[31] Laganà V et al (2022) Neuropsychiatric or behavioral and psychological symptoms of dementia (BPSD): focus on prevalence and natural history in Alzheimer’s disease and frontotemporal dementia. Front Neurol. 10.3389/fneur.2022.832199 35812082 10.3389/fneur.2022.832199 PMC9263122

[32] Hüsken JM, Halek M, Holle D, Dichter MN (2024) Prevalence of neuropsychiatric symptoms of people with dementia in long-term care units: a secondary analysis. Pflege 37(3):119–129. 10.1024/1012-5302/a000948 37409731 10.1024/1012-5302/a000948

[33] De Oliveira AM et al (2015) Nonpharmacological interventions to reduce behavioral and psychological symptoms of dementia: a systematic review. BioMed Res Int. 10.1155/2015/218980 26693477 10.1155/2015/218980 PMC4676992

[34] Gillis K et al (2023) Effect of need-based care on behavioural and psychological symptoms in residents with dementia and formal caregivers’ distress in nursing homes: a three-arm cluster randomized controlled trial. European Geriatr Med 14(5):1083–1096. 10.1007/s41999-023-00825-7 37405630 10.1007/s41999-023-00825-7

[35] Gillis K et al (2024) The impact of need-based care on formal caregivers’ wellbeing in nursing homes: a cluster randomized controlled trial. Int J Nurs Stud 150:104654. 10.1016/j.ijnurstu.2023.104654 38101268 10.1016/j.ijnurstu.2023.104654

